# Poster Session II - A281 CLINICAL OUTCOMES AND DRUG SUSTAINABILITY AFTER NON-MEDICAL SWITCH FROM USTEKINUMAB ORIGINATOR TO BIOSIMILARS IN INFLAMMATORY BOWEL DISEASE

**DOI:** 10.1093/jcag/gwaf042.281

**Published:** 2026-02-13

**Authors:** J Kritzinger, P Lakatos, G Kotrri, T Bessissow, I Candel, W Afif, A Bitton, H Nadeem, G Wild

**Affiliations:** McGill University, Montreal, QC, Canada; McGill University, Montreal, QC, Canada; McGill University, Montreal, QC, Canada; McGill University, Montreal, QC, Canada; McGill University, Montreal, QC, Canada; McGill University, Montreal, QC, Canada; McGill University, Montreal, QC, Canada; McGill University, Montreal, QC, Canada; McGill University, Montreal, QC, Canada

## Abstract

**Background:**

Biologic therapies have transformed the management of inflammatory bowel disease (IBD), yet their high cost poses substantial challenges for healthcare systems. Biosimilars offer a cost-effective alternative, with extensive evidence supporting the safety and efficacy of non-medical switching for infliximab and adalimumab. However, real-world data on ustekinumab biosimilars in IBD remain limited. Given increasing mandates for non-medical switches in Canada, evaluating clinical outcomes is critical to ensure patient safety and treatment sustainability. We hypothesized that switching from originator ustekinumab to a biosimilar would preserve clinical efficacy, safety, and drug persistence in patients with IBD.

**Aims:**

To evaluate clinical efficacy, drug sustainability, biomarker activity and adverse events (AEs) in IBD patients who underwent non-medical biosimilar switching from ustekinumab originator to a biosimilar.

**Methods:**

This was an observational study of consecutive IBD patients who underwent biosimilar switch. Disease activity, biomarkers, drug sustainability, and AEs were captured 8 weeks before switch, at the time of switch (baseline), 12 and 24 weeks after the switch.

**Results:**

81 patients were included [85.2% had Crohn’s disease (CD), the median age at inclusion: 42 years (IQR 29-61)]. Previous biological exposure was 82.7% and a dose optimization of the originator ustekinumab was performed in 63% before the switch. Drug sustainability at 12 and 24 weeks of switch was 96.3% and 95%, regardless of disease type or phenotype. The discontinuation rate was 4.9%. There was no significant difference in the rates of clinical remission at week 8 before switch, baseline, week 12, and 24 after switch: 87%, 85.9%, 84.3%, and 92.7%, p=NS. The biomarker activity was not significantly different for CRP, hemoglobin, albumin and fecal calprotectin (p=NS). All patients who stopped therapy after the non-medical switch needed a dose optimisation of the originator ustekinumab and had previous biological therapy prior to starting the ustekinumab originator.

**Conclusions:**

Despite prior biologic exposure and frequent dose escalation, switching to ustekinumab biosimilar showed stable efficacy, unchanged biomarkers, and high drug sustainability.

**Funding Agencies:**

None

